# Fiber Taper-Based Mach–Zehnder Interferometer for Ethanol Concentration Measurement

**DOI:** 10.3390/mi10110741

**Published:** 2019-10-31

**Authors:** Changrui Liao, Feng Zhu, Peng Zhou, Ying Wang

**Affiliations:** 1 Guangdong and Hong Kong Joint Research Centre for Optical Fibre Sensors, College of Physics and Optoelectronic Engineering, Shenzhen University, Shenzhen 518060, China; 2 Key Laboratory of Optoelectronic Devices and Systems of Ministry of Education and Guangdong Province, Shenzhen University, Shenzhen 518060, China

**Keywords:** optical fiber sensor, Mach-Zehnder interferometer, ethanol concentration measurement, femtosecond laser micromachining

## Abstract

We present a new type of fiber Mach–Zehnder interferometer based on a fiber taper and a pair of inner air bubbles for highly sensitive ethanol concentration measurement. The experimental results show there is a nonlinear relationship between the wavelength shift of the dip located near 1485 nm and the ethanol concentration but in the concentration range from 0.3 to 0.7 it can be seen as a linear response with a sensitivity of 28 nm/vol.

## 1. Introduction

Fiber Mach–Zehnder interferometer (MZI) is a type of useful sensing configuration, which has been widely studied in recent years. There are many ways for fiber MZI fabrication, which can be mainly divided into three categories. The first one is the sandwiched configuration, such as setting-off a short section of photonic crystal fiber (PCF) between two single mode fibers (SMFs) [1]; splicing two displaced SMFs after collapsing and splicing one section of PCF [2]; splicing two sections of multi-mode fibers (MMFs) in SMF [3] or replacing one section of MMF with a long period fiber grating (LPG) [4], to our knowledge, if these fiber MZIs are used to detect the refractive index of solution which is changed from 1.33~1.36, the highest sensitivity of sensors is ~450 nm/RIU. The second one employs some special fibers to generate optical path difference with the light propagating through the fiber [5,6,7,8,9,10,11]. The third one introduces some micro-structures in fiber to build fiber MZI by use of some micromachining method [12], when these fiber MZIs are used to detect the refractive index of solution, the sensitivity of sensors can reach up to ~10^4^ nm/RIU but this kind of devices are difficult to reuse. This paper will introduce a sensor which can achieve a balance between high sensitivity and structural integrity.

Ethanol plays an important role in our daily life, medical treatment, and scientific experiments. Therefore, the measurement of ethanol concentration is an active research topic. Many fiber optic sensors have been successfully used to measure ethanol concentration. For example, the ethanol concentration has been measured by coating gold film on polished fiber to excite surface plasmon resonance (SPR) [13], but this method is complex and costly. Some researchers used micro/nano-fiber Bragg-grating to measure the ethanol concentration [14], but the mechanical strength of this fiber is very weak. Alternatively, some researchers coated a layer of phenolic varnish film on a cone-shaped fiber surface. The fiber’s refractive index will change when in contact with ethanol, leading to an intensity change of the emitted light; thus, ethanol concentration can be characterized by light intensity [15]. However, this method is susceptible to environmental interference. In this paper, a fiber taper-based MZI was proposed for ethanol concentration measurement. The sensor has a compact and stable structure, and can be fabricated easily. By characterizing ethanol concentration via the wavelength change, this design improves the device’s reliability. The sensor consists of two inner air cavities and a section of taper. After being exposed at the end face of the fiber, femtosecond laser pulses introduce defects on the end face. During splicing, as the air is rapidly heated and expanded, a cavity forms in the fiber when the discharging ends and the air cools [16,17,18,19,20,21]. During fiber splicing, if the forces applied to the two fibers are opposite, the diameter of fibers near the fusing point will be smaller, forming a smooth tapered area. Based on these two technologies, an MZI sensor can be fabricated. As the effective refractive index corresponding to the mode in the fiber changes when the tapered section is immersed in liquids with different refractive indexes, the interference spectrum of the MZI will drift. The relationship between the concentration and the corresponding wavelength of the dip located near 1485 nm can be fitted by cubic curve. At the same time, it can be seen as a linear relationship when the concentration of alcohol solution is in the range of 0.3–0.7. Furthermore, the sensitivity reaches as high as 28 nm/vol, which is corresponding to 592.8 nm/RIU.

## 2. Methods

The fabrication of the device consists of three steps, the detailed process is as follows: 

Firstly, femtosecond laser pulses are applied to the center of end face of SMF, the laser power is set to 1 mW for the exposure.

**Figure 1:**
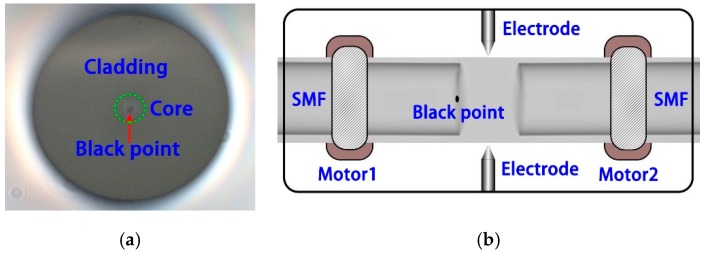
(**a**) Fiber end face ablated by femtosecond laser; (**b**) ablated single mode fibers (SMF) being spliced with the other normal SMF.

Secondly, the shutter is opened for 1s, then the beam will destroy the fiber end face. As a result, the area where the laser exposure will be ablated and looks like a black dot, as shown in Figure 1a. Actually, after this step, another ablated point exists under the end face because of the self-focusing effect. The laser pulse is visualized as subdivided into many thin intensity or power slices in time during the propagation. If its peak power is much higher than the critical power for self-focusing, the central slice at the peak of the pulse will self-focus at a distance *z_f_* from the beginning of the propagation in the medium given by [22,23]:

(1) $$z_{f}=\frac{0.376kr^{2}}{{\left[{\left(\sqrt{\frac{p}{p_{c}}}-0.825\right)}^{2}-0.0219\right]}^{1/2}}$$

*k* and *r* are the wave number and the radius of the beam profile at the 1/e level of intensity, respectively. The wavelength of laser beam is ~800 nm, *k* can be calculated by 2π/*λ* which is 7.854 μm^−1^. Due to the beam focused by 100× object lens, *r* is ~2 μm. *p* is the peak power of the slice which is ~1000 nJ, and *p_c_* is the threshold power which is ~100 nJ. If the range of *p*/*p_c_* is 1.01~10, finally, $z_{f}$ will located at 5.2~300 μm. Therefore, there is a refractive index modulation point on the end face of the fiber, and the other one at a certain distance below the end face.

Thirdly, the fabricated fiber is put into a fusion splicer, as shown in Figure 1b, under the tapering splicing mode, the parameters contain discharge power, discharge time, tapering length, and tapering speed which are set to standard, 2000 ms, 200 µm, and 45-bit, respectively.

**Figure 2:**
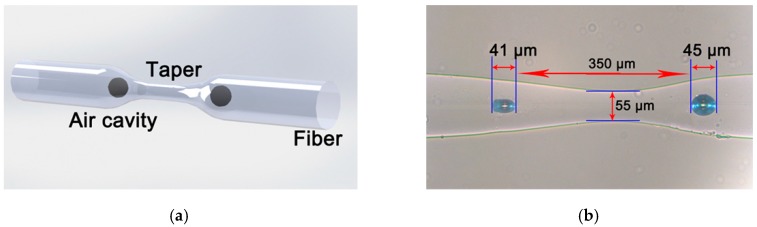
(**a**) Schematic diagram of fiber taper based Mach–Zehnder interferometer (MZI); (**b**) optical microscope image of one sample.

Finally, during the splicing, there will be an inner air bubble at each side of the taper. Actually, both refractive index modulation points are located on the fiber placed on the left side of Figure 1b but during the tapering the relative position between the electrodes and the splicing point shifts laterally so that the two air bubbles are located on both sides of the taper. Figure 2a shows the schematic diagram of this device, and Figure 2b is an optical microscope image of one sample. In this sample, the axial length of the tapered area is ~200 μm, the minimum radial diameter is ~55 μm, and the diameters of the two bubbles are ~41 μm and ~45 μm, respectively. Two samples with different geometric size have been fabricated with the parameters shown in Table 1.

## 3. Results and Discussion

**Figure 3:**
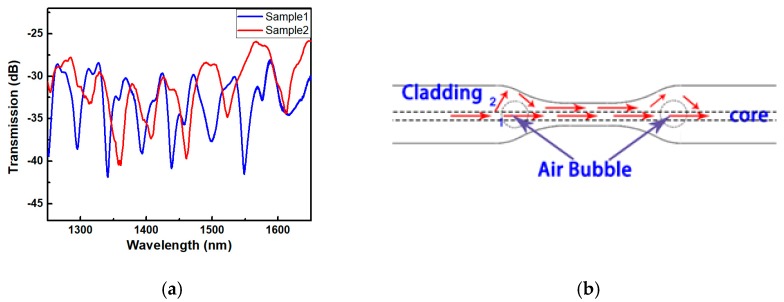
(**a**) Transmission spectra of two samples with different dimensions; (**b**) the principle of light transmission in the fiber.

Figure 3a shows the transmission spectra of these two samples. The principle for light transmission in the fiber is illustrated in Figure 3b. When the incident light is transmitted to the first bubble, part of the light passes through the bubble and continues to axially transmitted along the fiber core, and the other part of the light is transmitted in the cladding for a short distance, passing through the tapered section, and is returned to the core after passing by the second bubble. In this way, two light beams with a certain optical path difference are formed, causing interference.

Evidently, the device meets the characteristics of MZI and the free spectral range (FSR) can be calculated by the following formula [8,10,17]:

(2) $$\mathrm{FSR}=\frac{\lambda^{2}}{\Delta n_{b}\Delta L_{b}-\Delta n_{t}\Delta L_{t}}=\frac{\lambda^{2}}{\Delta \mathrm{OPD}}$$

where *λ* is the interference wavelength, $\Delta n_{b}$ and $\Delta n_{t}$ are the effective refractive index difference between fiber core and air and taper area, respectively; $\Delta L_{b}$ and $\Delta L_{t}$ denote the geometrical length passing through the air cavity and the length of taper area, respectively. Furthermore, ΔnΔL can be replaced by ΔOPD (optical path difference). ΔOPD is mainly determined by the size of the air cavity and the refractive index of the taper and the size of air cavity is the predominant contributor. The FSR is shown in Figure 3a. The median of two wavelengths corresponding to two dips is taken to be the central wavelength, the refractive index of the air is 1 and the refractive index of the cladding is 1.44. All these values were substituted into Equation (2) to calculate the average geometrical lengths of the two samples, which are 91 μm and 78 μm, respectively. They are approximately equal to the total diameters of the two bubbles in each sample.

In the test the environmental temperature is kept unchanged and the fiber MZI is fixed between two fiber holders to ensure the strain applied on the fiber is a constant. When the tapered section is immersed in liquids with different refractive index, the liquid modifies the effective refractive index of the high-order mode transmitted in the cladding. As a result, the optical path of Light Beam 2 transmitted in the cladding is increases, while that of Light Beam 1, transmitted along the fiber core, remains basically the same, the difference between optical paths of the two light beams increases, which thereby leads to spectral drift and the reduction of FSR, reflected by a red shift in the spectrum.

**Figure 4:**
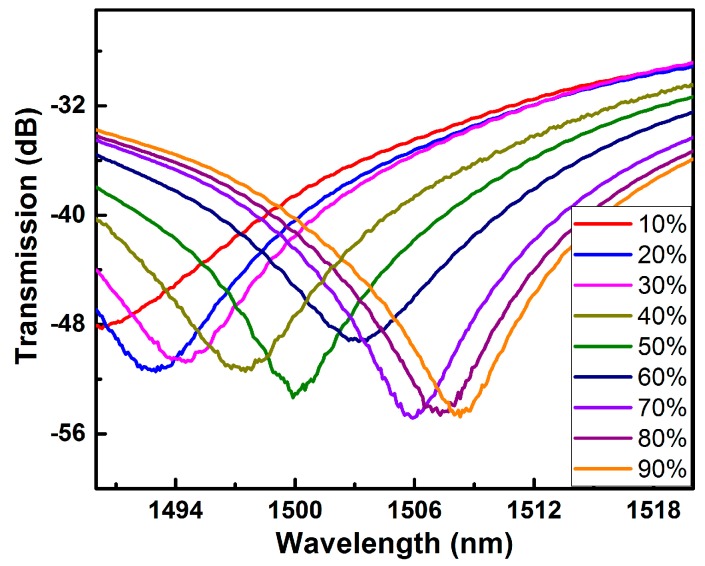
Evolution of transmission spectra of Sample 2 in ethanol aqueous solutions with different concentrations.

Ethanol aqueous solutions with different volume fractions have different concentrations, and consequently, different refractive indices. Thus, when the samples are immersed in aqueous ethanol solutions with different volume fractions, the change in the volume fraction of the solution can be reflected by the change of one specific dip in the MZI spectrum. In the test, distilled water with different volumes and an equal volume of industrial ethanol were mixed into aqueous ethanol solutions with different volume fractions, and then Sample 2 was soaked in these formulated solutions. Figure 4 shows the change of the drift of a dip located near 1485 nm in Sample 2 in aqueous ethanol solutions with different volume fractions.

As shown in Figure 4, with the increase of the volume fraction of ethanol, the dip drifts towards a longer wavelength, but the variation of the wavelength corresponding to the dip decreases as the volume fraction increases. Therefore, the change in wavelength does not have a linear relationship with the volume fraction of the aqueous ethanol solution, for the refractive index of the solution does not vary linearly with the volume fraction [13,14,15].

**Figure 5:**
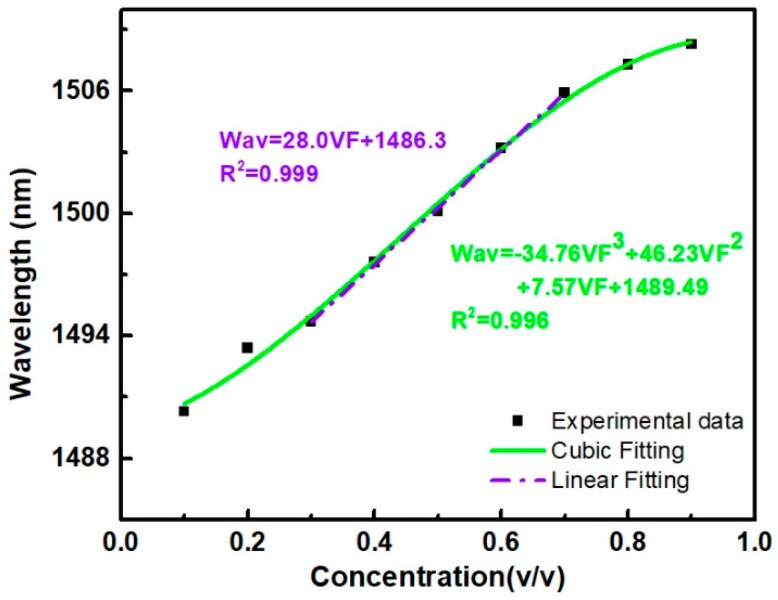
Relationship between the volume fraction of ethanol aqueous solution and the wavelength of the dip near 1485 nm.

Figure 5 shows the relationship between the volume fraction of the ethanol aqueous solution and the wavelength corresponding to the dip located near 1485 nm. The black squares in Figure 5 represent the data points measured in the test. According to the curve fitting results, the error value is low when cubic curve fitting is used.

The green curve in the figure is the cubic curve obtained based on the experimental data, and the formula of the curve is:

(3) $$\mathrm{Wav}=34.8{\mathrm{VF}}^{3}+46.2{\mathrm{VF}}^{2}+7.6\mathrm{VF}+1489.5$$

where Wav denotes the wavelength corresponding to the dip being monitored (in nanometers), and VF is the volume fraction of the ethanol aqueous solution. It is found that the dip wavelength shift is linearly with the concentration variation, when the concentration is in the range of 0.3–0.7, as shown in Figure 5. The purple dot dash line can be expressed as:

(4) Wav=28.0VF+1486.3

From Equation (4), it is known that the sensitivity of the sensor can be reached up to 2.8 nm/vol. The relationship between the ethanol concentration and its refractive index has been measured and summarized in Table 2.

**Figure 6:**
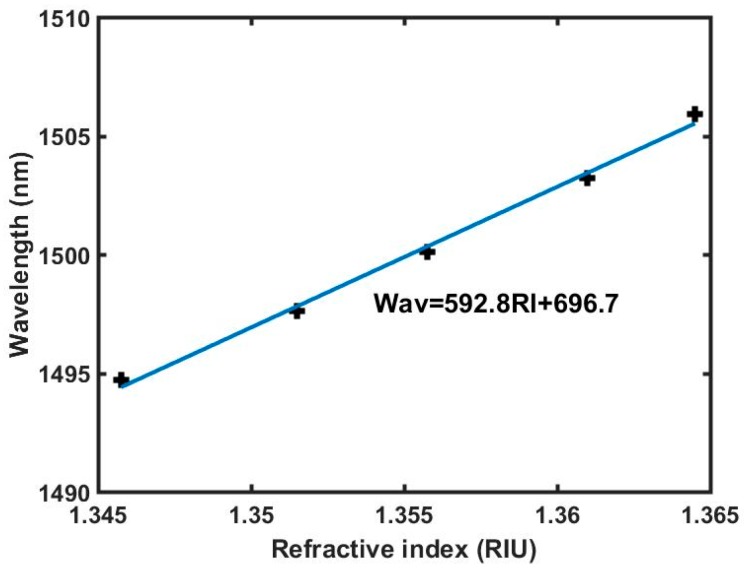
Relationship between the dip wavelength and the refractive index of ethanol solution.

Combined with the experimental data, the relationship between the dip wavelength and the refractive index of ethanol solution is illustrated in Figure 6, where the black cross is experimental data and the blue line is the linear fitting result. The refractive index sensitivity is calculated as 592.8 nm/RIU, which is higher than those of most fiber taper-based MZIs.

## 4. Conclusions

In this paper, a simple and convenient method for fabricating MZIs was introduced. Specifically, the fiber end face is ablated by femtosecond laser pulses, and then tapered splicing was performed by a commercial fiber splicer. By using the above method, a fiber MZI which consists of two inner air cavities and a taper in between was successfully fabricated. This fiber MZI can be used for ethanol concentration measurement and there is a nonlinear relationship between the wavelength shift of the dip located near 1485 nm and the ethanol concentration but in the concentration range from 0.3 to 0.7 it can be seen as a linear response with a sensitivity of 28 nm/vol corresponding to 592.8 nm/RIU, which is higher than kindred sensors.

## Figures and Tables

**Table 1:** Geometric dimension of two samples.

Device Parameters	Sample 1	Sample 2
Taper length (μm)	200	350
Diameter of Bubble 1 (μm)	69	41
Diameter of Bubble 2 (μm)	29	45
Total bubble diameters (μm)	98	86

**Table 2:** The measured refractive index of ethanol solution with different concentrations.

Concentration	RI_1_	RI_2_	RI_3_	RI_4_	Average RI
30%	1.3462	1.3457	1.3456	1.3455	1.3458
40%	1.3515	1.3518	1.3514	1.3513	1.3515
50%	1.3558	1.3559	1.3560	1.3555	1.3558
60%	1.3608	1.3611	1.3611	1.3610	1.3610
70%	1.3646	1.3645	1.3645	1.3644	1.3645
